# Supplementary material for: EGFAFS: A Novel Feature Selection Algorithm Based on Explosion Gravitation Field Algorithm
Source: Entropy (Basel). 2022 Jun 25;24(7):873. doi: 10.3390/e24070873 (PMC9322764; doi:10.3390/e24070873)
Supplement: Supplementary file 1 [file entropy-24-00873-s001.zip › entropy-1762464-supplementary.pdf]

Supplementary

Table S1 shows the results of EGFAFS with different sizes of subsets. Specifically, the size of the feature subsets is set to 30, 50, 100, and 150.

Table S1. Comparison of EGFAFS with different sizes of subsets

Dataset	size	ACC	F1	Recall	PRE	MCC	AP	AUC
HNSC	30	**1.0000**	**1.0000**	**1.0000**	**1.0000**	**1.0000**	**1.0000**	**1.0000**
	50	0.9909	0.9090	0.8333	**1.0000**	0.9085	**1.0000**	**1.0000**
	100	**1.0000**	**1.0000**	**1.0000**	**1.0000**	**1.0000**	**1.0000**	**1.0000**
	150	0.9909	0.9090	0.8333	**1.0000**	0.9085	0.9583	0.9967
LIHC	30	**0.9882**	**0.9600**	**1.0000**	**0.9230**	**0.9541**	0.9935	0.9988
	50	**0.9882**	**0.9600**	**1.0000**	**0.9230**	**0.9541**	**1.0000**	**1.0000**
	100	0.9629	0.8571	**1.0000**	0.7500	0.8477	**1.0000**	**1.0000**
	150	0.9764	0.9166	0.9166	0.9166	0.9029	0.9790	0.9965
LUAD	30	0.9826	0.9166	0.8461	**1.0000**	0.9999	**1.0000**	**1.0000**
	50	**1.0000**	**1.0000**	**1.0000**	**1.0000**	**1.0000**	0.9999	**1.0000**
	100	**1.0000**	**1.0000**	**1.0000**	**1.0000**	**1.0000**	**1.0000**	**1.0000**
	150	**1.0000**	**1.0000**	**1.0000**	**1.0000**	**1.0000**	**1.0000**	**1.0000**
LUSC	30	**1.0000**	**1.0000**	**1.0000**	**1.0000**	**1.0000**	**1.0000**	**1.0000**
	50	**1.0000**	**1.0000**	**1.0000**	**1.0000**	**1.0000**	**1.0000**	**1.0000**
	100	0.9859	0.8000	**1.0000**	0.6666	0.8105	**1.0000**	**1.0000**
	150	**1.0000**	**1.0000**	**1.0000**	**1.0000**	**1.0000**	**1.0000**	**1.0000**
PRAD	30	0.9727	0.8235	**0.8750**	0.7777	0.8104	0.9149	0.9865
	50	**0.9909**	**0.9333**	**0.8750**	**1.0000**	**0.9308**	0.9416	0.9914
	100	0.9428	0.7142	0.7142	0.7142	0.6825	0.7382	0.9455
	150	0.9818	0.8750	**0.8750**	0.8750	0.8651	**0.9464**	**0.9926**
STAD	30	0.9756	0.8000	0.6666	**1.0000**	0.8059	0.7916	0.9583
	50	0.9634	0.6666	0.5000	**1.0000**	0.6935	0.8854	0.9817
	100	**0.9878**	**0.9090**	**0.8333**	**1.0000**	**1.0000**	**0.9069**	0.9444
	150	0.9538	0.7272	0.6666	0.8000	0.7057	0.8773	**0.9858**
THCA	30	**1.0000**	**1.0000**	**1.0000**	**1.0000**	**1.0000**	**1.0000**	**1.0000**
	50	**1.0000**	**1.0000**	**1.0000**	**1.0000**	**1.0000**	**1.0000**	**1.0000**
	100	0.9912	0.9600	0.9230	**1.0000**	0.9560	**1.0000**	**1.0000**
	150	0.9780	0.8888	0.8888	0.8888	0.8766	0.9888	0.9986
UCEC	30	0.9913	0.9333	0.8750	**1.0000**	0.9311	**1.0000**	**1.0000**
	50	**1.0000**	**1.0000**	**1.0000**	**1.0000**	**1.0000**	**1.0000**	**1.0000**
	100	0.9913	0.9411	**1.0000**	0.8888	0.9384	**1.0000**	**1.0000**
	150	0.9913	0.9333	0.8750	**1.0000**	0.9311	**1.0000**	**1.0000**

Table S2 shows the results of EGFAFS with different sizes of the feature recommended pool. Specifically, the size of the feature recommended pool is set to 100, 300, and 500, respectively.

Table S2. Comparison of the performance with different sizes of the feature recommended pool

Dataset	size	ACC	F1	Recall	PRE	MCC	AP	AUC
HNSC	100	0.9636	0.6666	0.6666	0.6666	0.6474	0.8333	0.9727
	300	**0.9909**	**0.9090**	0.8333	**1.0000**	**0.9085**	**1.0000**	**1.0000**
	500	0.9818	0.8333	**0.8333**	0.8333	0.8333	0.9761	0.9983
LIHC	100	0.9764	0.9166	0.9166	0.9166	0.9029	0.9935	0.9988
	300	**0.9882**	**0.9600**	**1.0000**	**0.9230**	**0.9541**	**1.0000**	**1.0000**
	500	0.9647	0.8799	0.9166	0.8461	0.8602	0.8461	0.8602
LUAD	100	**1.0000**	**1.0000**	**1.0000**	**1.0000**	**1.0000**	**0.9999**	**1.0000**
	300	**1.0000**	**1.0000**	**1.0000**	**1.0000**	**1.0000**	**0.9999**	**1.0000**
	500	0.9826	0.9166	0.8461	**1.0000**	0.9109	0.9945	0.9992
LUSC	100	**1.0000**	**1.0000**	**1.0000**	**1.0000**	**1.0000**	**1.0000**	**1.0000**
	300	**1.0000**	**1.0000**	**1.0000**	**1.0000**	**1.0000**	**1.0000**	**1.0000**
	500	**1.0000**	**1.0000**	**1.0000**	**1.0000**	**1.0000**	**1.0000**	**1.0000**
PRAD	100	0.9636	0.75	0.75	0.75	0.7303	0.7940	0.9718
	300	**0.9909**	**0.9333**	**0.8750**	**1.0000**	**0.9308**	**0.9416**	**0.9914**
	500	0.9545	0.6666	0.625	0.7142	0.6440	0.8588	0.9803
STAD	100	0.9512	0.6	0.5	0.75	0.5885	0.9334	0.9916
	300	0.9634	0.6666	0.5000	**1.0000**	0.6935	0.8854	0.9817
	500	**0.9878**	**0.9090**	**0.8333**	**1.0000**	**0.9069**	**1.0000**	**1.0000**
THCA	100	**1.0000**	**1.0000**	**1.0000**	**1.0000**	**1.0000**	**1.0000**	**1.0000**
	300	**1.0000**	**1.0000**	**1.0000**	**1.0000**	**1.0000**	**1.0000**	**1.0000**
	500	0.9561	0.8275	0.9230	0.75	0.8084	0.9334	0.9916
UCEC	100	0.9913	0.9333	0.875	**1.0000**	0.9311	0.9861	0.9861
	300	**1.0000**	**1.0000**	**1.0000**	**1.0000**	**1.0000**	**1.0000**	**1.0000**
	500	0.9827	0.8571	0.75	**1.0000**	0.8581	**1.0000**	**1.0000**

Table S3-S10 represent the detailed information of comparison results of our EGFAFS and eight well-known FS methods (GA, PSO, SA, DE, EGSG, Boruta, HSICLasso, and DNN-FS) on eight gene expression datasets (HNSC, LIHC, LUAD, LUSC, PRAD, STAD, THCA, and UCEC).

Table S3. Comparison of different methods on HNSC

Method	ACC	F1	Recall	PREF	MCC	AP	AUC
GA	0.9818	0.8333	**0.8333**	0.8333	0.8237	0.9761	0.9983
PSO	0.9818	0.8333	**0.8333**	0.8333	0.8237	0.9305	0.9951
SA	0.9736	0.8695	0.7692	**1.0000**	0.8643	0.9796	0.9969
DE	0.9818	0.8333	**0.8333**	0.8333	0.8237	0.8972	0.9935
EGSG	0.9454	0.2500	0.1666	0.5000	0.2669	0.4039	0.9214
Boruta	0.9545	0.4444	0.3333	0.6667	0.4513	0.8357	0.9903
HSICLasso	**0.9909**	**0.9090**	**0.8333**	1.0000	**0.9085**	0.9444	0.9951
DNN-FS	0.9727	0.7272	0.6666	0.8000	0.7163	0.8857	0.9903
EGFAFS	**0.9909**	**0.9090**	**0.8333**	**1.0000**	**0.9085**	**1.0000**	**1.0000**

Table S4. Comparison of different methods on LIHC

Method	ACC	F1	Recall	PRE	MCC	AP	AUC
GA	0.9529	0.8181	0.7500	0.9000	0.7957	0.9537	0.9908
PSO	0.9647	0.8695	0.8333	0.9090	0.8502	0.9735	0.9954
SA	0.9529	0.8181	0.7500	0.9000	0.7957	0.9437	0.9908
DE	0.9647	0.8799	0.9166	0.8461	0.8602	0.9676	0.9942
EGSG	0.9411	0.7368	0.5833	**1.0000**	0.7388	0.8952	0.9691
Boruta	0.9647	0.8696	0.8333	0.9090	0.8503	0.9579	0.9579
HSICLasso	0.9529	0.8181	0.7500	0.9000	0.7957	0.9614	0.9942
DNN-FS	0.9764	0.9090	0.8333	**1.0000**	0.9006	**1.0000**	**1.0000**
EGFAFS	**0.9882**	**0.9600**	**1.0000**	0.9230	**0.9541**	**1.0000**	**1.0000**

Table S5. Comparison of different methods on LUAD

Method	ACC	F1	Recall	PRE	MCC	AP	AUC
GA	0.9913	0.9600	0.9230	**1.0000**	0.9560	**0.9999**	**1.0000**
PSO	0.9913	0.9600	0.9230	**1.0000**	0.9560	0.9945	0.9992
SA	0.9913	0.9600	0.9230	**1.0000**	0.9560	**0.9999**	**1.0000**
DE	0.9913	0.9600	0.9230	**1.0000**	0.9560	**0.9999**	**1.0000**
EGSG	0.9304	0.5555	0.3846	**1.0000**	0.5971	**0.9999**	**1.0000**
Boruta	0.9913	0.9600	0.9230	**1.0000**	0.9561	0.9945	0.9992
HSICLasso	**1.0000**	**1.0000**	**1.0000**	**1.0000**	**1.0000**	**0.9999**	**1.0000**
DNN-FS	0.9826	0.9166	0.8461	**1.0000**	0.9109	0.9897	0.9984
EGFAFS	**1.0000**	**1.0000**	**1.0000**	**1.0000**	**1.0000**	**0.9999**	**1.0000**

Table S6. Comparison of different methods on LUSC

Method	ACC	F1	Recall	PRE	MCC	AP	AUC
GA	**1.0000**	**1.0000**	**1.0000**	**1.0000**	**1.0000**	**1.0000**	**1.0000**
PSO	**1.0000**	**1.0000**	**1.0000**	**1.0000**	**1.0000**	**1.0000**	**1.0000**
SA	**1.0000**	**1.0000**	**1.0000**	**1.0000**	**1.0000**	**1.0000**	**1.0000**
DE	**1.0000**	**1.0000**	**1.0000**	**1.0000**	**1.0000**	**1.0000**	**1.0000**
EGSG	0.9545	0.7368	0.5833	**1.0000**	0.7449	0.9692	0.9940
Boruta	0.9818	0.9091	0.8333	**1.0000**	0.9037	0.9936	0.9991
HSICLasso	**1.0000**	**1.0000**	**1.0000**	**1.0000**	**1.0000**	**1.0000**	**1.0000**
DNN-FS	**1.0000**	**1.0000**	**1.0000**	**1.0000**	**1.0000**	**1.0000**	**1.0000**
EGFAFS	**1.0000**	**1.0000**	**1.0000**	**1.0000**	**1.0000**	**1.0000**	**1.0000**

Table S7. Comparison of different methods on PRAD

Method	ACC	F1	Recall	PRE	MCC	AP	AUC
GA	0.9727	0.7999	0.7500	0.8571	0.7874	0.8638	0.9595
PSO	0.9727	0.7999	0.7500	0.8571	0.7874	0.9208	**0.9914**
SA	0.9727	0.7999	0.7500	0.8571	0.7874	0.9184	**0.9914**
DE	0.9636	0.7777	0.8750	0.7000	0.7638	0.9144	**0.**9901
EGSG	0.9545	0.6153	0.5000	0.8000	0.6111	0.6267	0.9644
Boruta	0.9455	0.4000	0.2500	**1.0000**	0.4859	0.7139	0.9228
HSICLasso	0.9636	0.7500	0.7500	0.7500	0.7304	0.7939	0.9730
DNN-FS	0.9636	0.7500	0.7500	0.7500	0.7303	0.8039	0.9362
EGFAFS	**0.9909**	**0.9333**	**0.8750**	1.0000	**0.9308**	**0.9416**	**0.9914**

Table S8. Comparison of different methods on STAD

Method	ACC	F1	Recall	PRE	MCC	AP	AUC
GA	0.9512	0.5000	0.3333	**1.0000**	0.5627	0.8284	0.9802
PSO	**0.9634**	**0.6666**	**0.5000**	**1.0000**	**0.6935**	0.6502	0.9232
SA	**0.9634**	**0.6666**	**0.5000**	**1.0000**	**0.6935**	0.8310	0.9714
DE	**0.9634**	**0.6666**	**0.5000**	**1.0000**	**0.6935**	0.8757	**0.9868**
EGSG	0.9512	0.5000	0.3333	**1.0000**	0.5627	0.6400	0.9407
Boruta	**0.9634**	**0.6666**	**0.5000**	**1.0000**	**0.6935**	0.7973	0.9714
HSICLasso	0.9146	0.2222	0.1666	0.3333	0.1946	0.6092	0.9627
DNN-FS	0.9390	0.4444	0.3333	0.6666	0.4441	0.7098	0.9714
EGFAFS	**0.9634**	**0.6666**	**0.5000**	**1.0000**	**0.6935**	**0.8854**	0.9817

Table S9. Comparison of different methods on THCA

Method	ACC	F1	Recall	PRE	MCC	AP	AUC
GA	**1.0000**	**1.0000**	**1.0000**	**1.0000**	**1.0000**	0.9999	**1.0000**
PSO	0.9912	0.9600	0.9230	1.0000	0.9560	0.9999	1.0000
SA	**1.0000**	**1.0000**	**1.0000**	**1.0000**	**1.0000**	0.9999	**1.0000**
DE	**1.0000**	**1.0000**	**1.0000**	**1.0000**	**1.0000**	0.9999	**1.0000**
EGSG	0.9561	0.7619	0.6153	**1.0000**	0.7657	0.9323	0.9878
Boruta	0.9912	0.9600	0.9231	**1.0000**	**1.0000**	0.9897	0.9984
HSICLasso	0.9824	0.9166	0.8461	**1.0000**	0.9108	0.9999	**1.0000**
DNN-FS	0.9824	0.9166	0.8461	**1.0000**	0.9108	0.9999	**1.0000**
EGFAFS	**1.0000**	**1.0000**	**1.0000**	**1.0000**	**1.0000**	**1.0000**	**1.0000**

Table S10. Comparison of different methods on UCEC

Method	ACC	F1	Recall	PRE	MCC	AP	AUC
GA	0.9827	0.8750	0.7500	**1.0000**	0.8581	0.9861	0.9988
PSO	0.9827	0.8750	0.8750	0.8750	0.8657	0.9704	0.9976
SA	**1.0000**	**1.0000**	**1.0000**	**1.0000**	**1.0000**	**1.0000**	**1.0000**
DE	0.9913	0.9333	0.8750	**1.0000**	0.9311	**1.0000**	**1.0000**
EGSG	0.9655	0.6666	0.5000	**1.0000**	0.6943	0.8293	0.9814
Boruta	0.9914	0.9333	0.875	**1.0000**	0.9311	0.9306	0.9884
HSICLasso	0.9827	0.8750	0.8750	0.8750	0.8657	0.9861	0.9988
DNN-FS	0.9827	0.8571	0.7500	**1.0000**	0.8581	**1.0000**	**1.0000**
EGFAFS	**1.0000**	**1.0000**	**1.0000**	**1.0000**	**1.0000**	**1.0000**	**1.0000**

Table S11 shows the distribution of genes found by nine FS methods (GA, SA, PSO, ED, EGSG, Boruta, HSICLasso, DNN-FS, and EGFAFS) on HNSC.

Table S11. The distribution of genes found by nine FS methods on HNSC

**Names**	**Total**	**Genes**
DE, EGFAFS, GA	1	KDM1A
DE, EGSG, PSO	1	TNMD
DE, GA, PSO	1	CEACAM1
DE, GA, SA	1	ERFE
DE, PSO, SA	1	WDR53
DE, HSICLasso, SA	2	FMO2, CAB39L
EGFAFS, GA, SA	1	TK1
EGSG, HSICLasso, PSO	1	ADIPOQ
Boruta, EGFAFS	1	FHOD1
Boruta, EGSG	1	SULT1C3
DE, EGFAFS	2	MORC2, WNT2
DE, GA	2	UHRF1, MED30
DE, PSO	2	IKBIP, DCBLD1
DE, HSICLasso	3	SHCBP1, COBL, BARX2
EGFAFS, GA	1	FOXD1
EGFAFS, PSO	1	VASP
EGFAFS, SA	5	NDRG2, COL5A2, P3H1, BMP1, MMP9
EGFAFS, HSICLasso	4	RRAGD, COLGALT1, FAM3D, ACADL
EGSG, GA	1	FAM71A
EGSG, PSO	1	C6
EGSG, HSICLasso	3	OTC, KCNC2, SMR3B
GA, SA	8	SLC27A6, CBX3, GJC1, UBL3, CCM2, ZNF707, HSD17B6, MMP12
GA, HSICLasso	4	HMGCS2, KRT13, TGM3, PLIN1
PSO, SA	1	NEU1
HSICLasso,	3	EMP1, ADH1B, ATP6V0A4

SA		
Boruta	28	NANOGP8, HOXB1, WDR24, CASKIN2, TPM4, DGKG, SF3A1, CD5L, CDC42EP2, PPID, FMO4, SLITRK5, C7orf33, OR4A47, APTX, SCT, ADGRG2, ASB6, WDR72, INTS8, EXOSC8, GUF1, CD247, CYP51A1, GLP1R, ZNF558, OR56A4, TP53TG5
DE	30	C1orf112, CKS1B, UBE2Z, CEP131, TNFRSF4, SORCS1, HMGB3, BMP8A, SPAG5, RHEBL1, GINS1, HOXC11, COL6A3, CCN4, ITGA6, HOXD10, HOXC4, CFAP251, QARS1, NDC80, GAST, SCG5, HSP90B1, SLC52A2, CLEC3B, EVPL, CAMK2N2, ARTN, CAPN5, SH2D2A
DNN-FS	50	L3HYPDH, NADK, NMUR1, C1S, ARHGAP32, CCDC110, STXBP2, SMIM12, ZNF208, GPR179, CYB561, SCAMP4, ZNF491, TRAK1, CTSG, KRTAP9-8, NFIB, SH3RF1, VEZT, C10orf71, PGGT1B, SREBF1, DTX3L, SLC18A2, CD300LD, ADGRE2, NR1D2, CEPT1, TLX2, TEPP, C2orf80, APOL1, RAD9A, STARD4, MYC, FERD3L, TMEM217, ANP32B, TM4SF19-DYNLT2B, RAD50, NUDCD1, HRH1, CST5, GABRR1, LCE4A, TNFRSF14, OSBPL9, UBQLN2, AMZ1, ANKRD65
EGFAFS	34	KIF26B, RNASEH2A, FOXD2, CCL11, HOMER3, ZNF114, MTFR2, MINDY1, OCIAD2, ABL2, LOXL2, CCNF, KPNA2, FAM107A, TEDC2, KIF2C, GPX3, DNAH17, ORC6, UBE2C, SMS, COL5A1, PMEPA1, CHST11, TGFB1, RFC4, TTK, PSMC3IP, SOCS1, PKMYT1, B3GAT3, NEK2, ERF, NAT14
EGSG	42	SMR3A, MOGAT1, LIPF, ANGPTL5, HCN1, TMOD4, CABS1, LHCGR, KRTAP1-3, BPIFA2, SLC5A7, NPY2R, SCGN, FIGLA, PABPC1L2A, PCDH20, GSTA5, OPRPN, OTOR, CSN2, ANKRD20A3P, C1orf87, KRTAP3-2, FTMT, CLDN25, LRRTM1, TEX26, FAM47E-STBD1, SPINK8, PPDPFL, CRISP2, WFDC6, PABPC1L2B, AMELY, SCGB1D2, LACRT, KRTAP1-1, MRGPRE, SERTM1, C14orf180, RPRML, FCN2
GA	23	ZFP92, ITGA5, HOXD8, EXT1, EFNB1, CERS5, KRT4, ACD, HDGF, ADAMTSL2, CDCA4, C18orf54, OIP5, PLOD3, GGTA1, TPPP2, H2AC11, CENPO, KIF14, NRIP3, CDHR2, HTR1D, ESM1
PSO	25	NPY5R, PDE6A, MOGS, SERPINE1, DTL, ALG3, SLC13A2, TCP11L1, CACNB4, BST2, BIRC5, MUCL3, NOX4, CRYBG2, IL36A, IFI30, MTBP, FUCA2, MTHFD1L, FSD1, TPBG, TROAP, SLC26A6, IGSF8, HJURP
SA	28	QSOX2, TMPRSS11B, BCL2L12, ADAM12, AP2M1, HOXD11, NRM, SERPINH1, PARP12, STX1A, PTK7, CDC25B, NUCB2, MMP14, HOXC8, COL10A1, HOXC9, GRIN2D, MRGBP, HOXD9, IBSP, MFAP2, COL13A1, CFTR, CDCA5, ISG15, PRIM2, MMP11
HSICLasso	30	PRH2, MYZAP, TPT1, ARSF, TMEM132C, PEBP4, UPK1A, DLG2, ENDOU, DYNAP, KRT36, RORC, CIDEC, NRG2, MT1A, BLOC1S3, GPRIN1, SLC38A3, GPD1, CSRNP1, KRT84, MYOC, SLC38A7, KRT78, ADH4, CRISP3, PLIN5, ZNF200, ATP6V1C1, PAIP2B

Figure S1-S8 show the degrees of the genes (features) selected by EGFAFS in differential co-expression networks for eight datasets (HNSC, LIHC, LUAD, LUSC, PRAD, STAD, THCA, and UCEC).

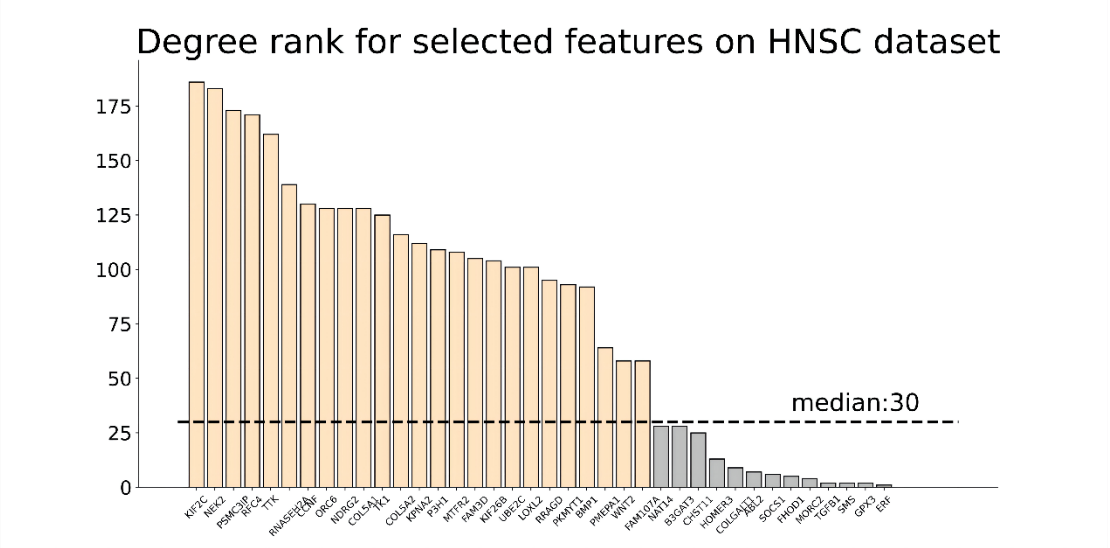

Figure S1. The degree of the features selected by EGFAFS for LIHC dataset in the differential co-expression network.

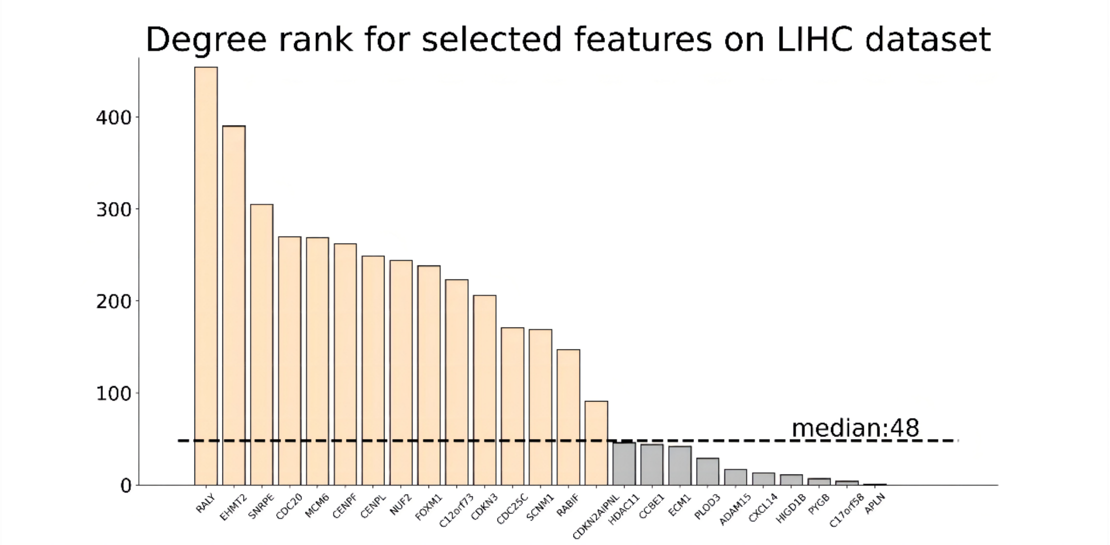

Figure S2. The degree of the features selected by EGFAFS for LIHC dataset in the differential co-expression network.

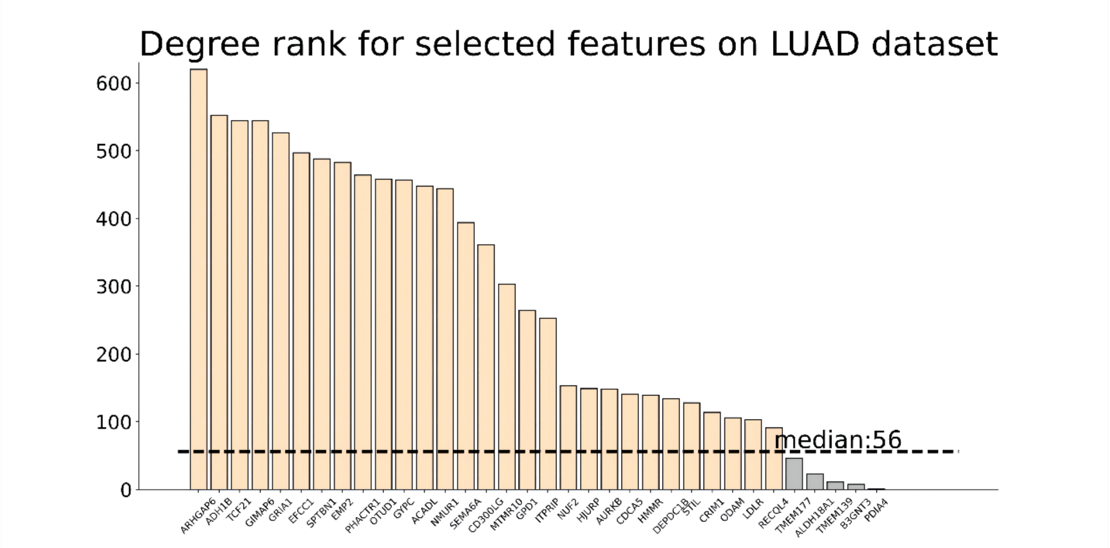

Figure S3. The degree of the features selected by EGFAFS for LUAD dataset in the differential co-expression network.

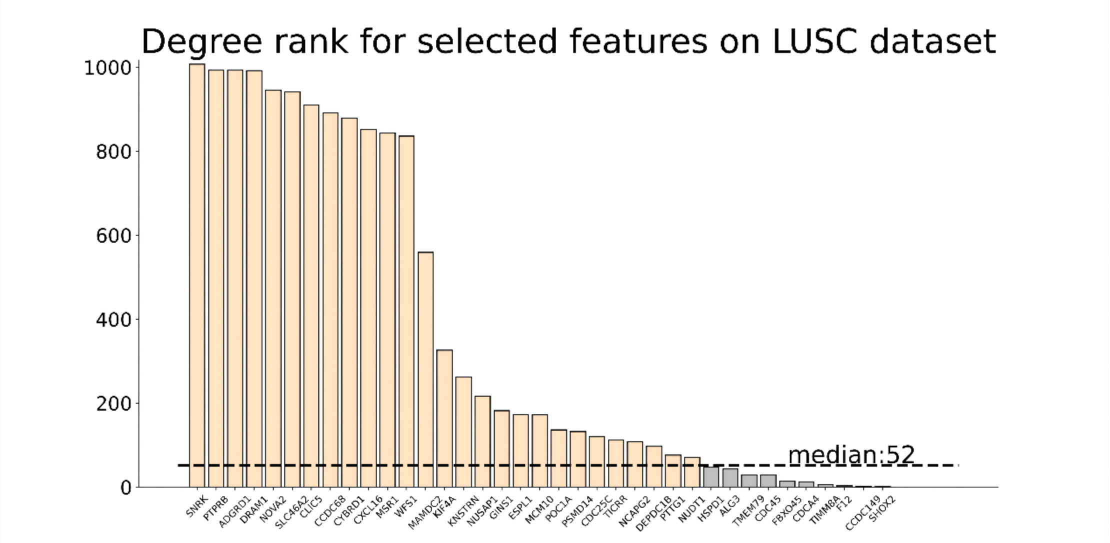

Figure S4. The degree of the features selected by EGFAFS for LUSC dataset in the differential co-expression network.

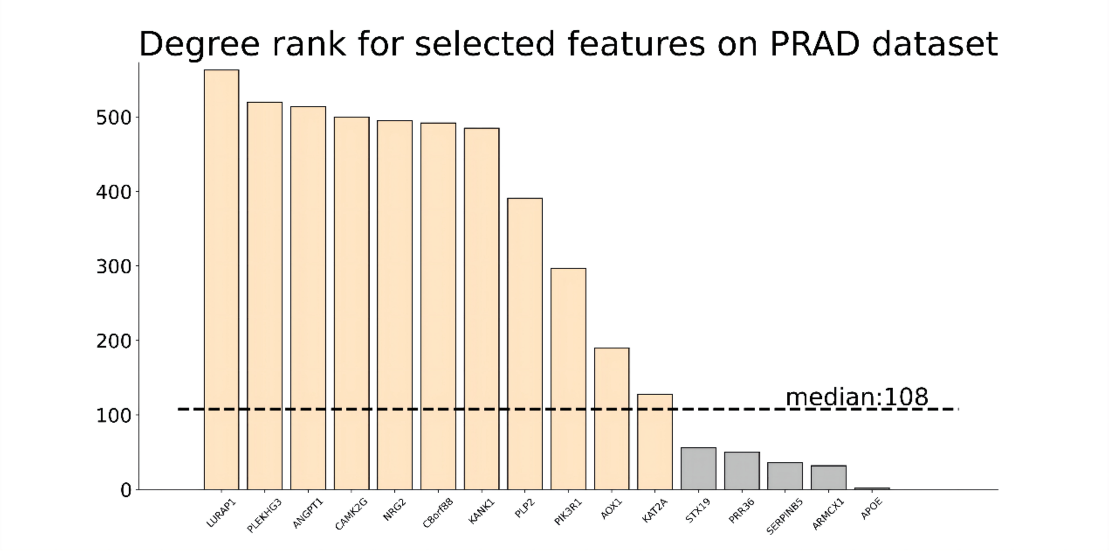

Figure S5. The degree of the features selected by EGFAFS for PRAD dataset in the differential co-expression network.

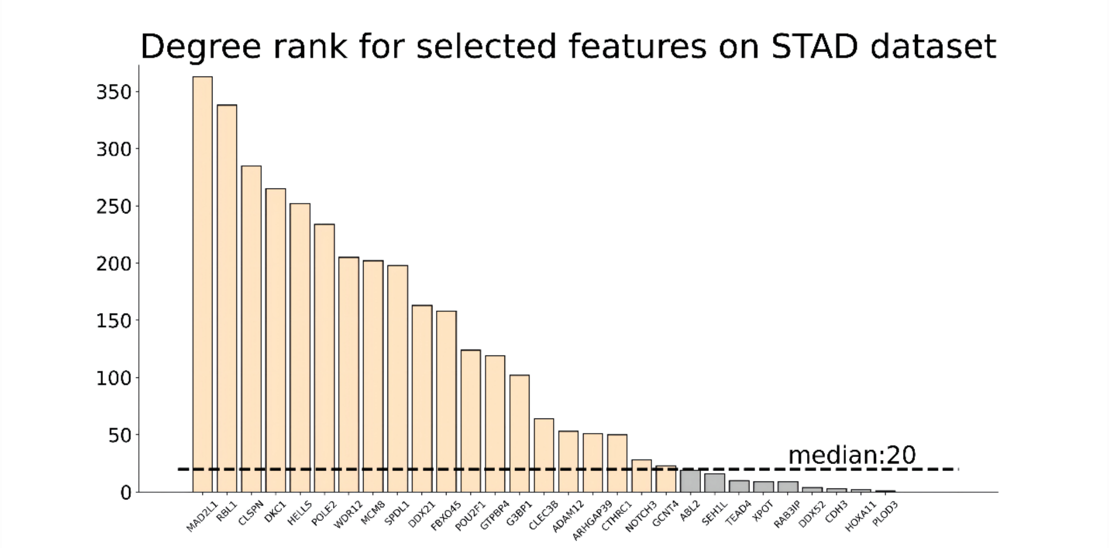

Figure S6. The degree of the features selected by EGFAFS for STAD dataset in the differential co-expression network.

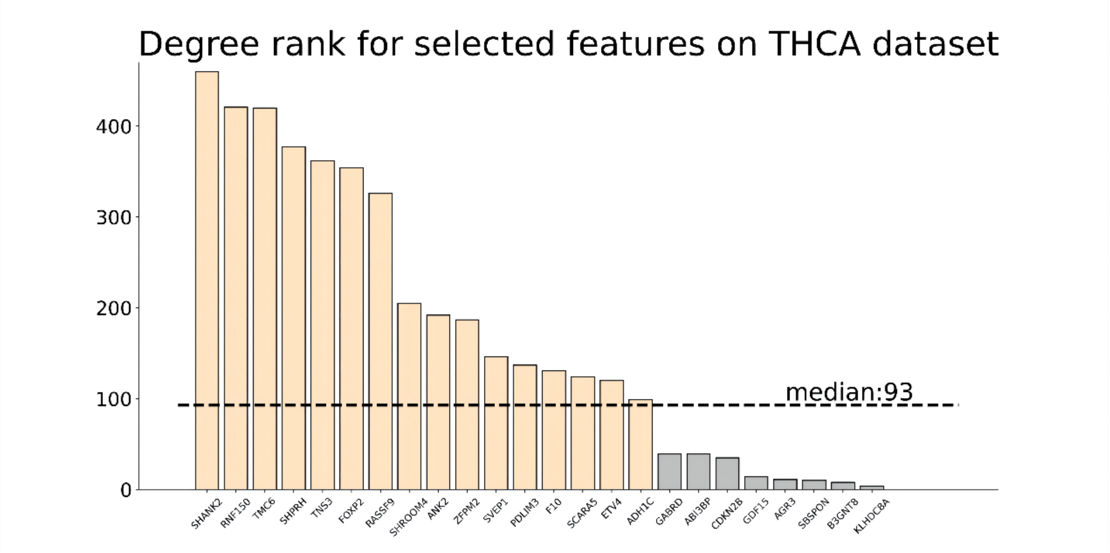

Figure S7. The degree of the features selected by EGFAFS for THCA dataset in the differential co-expression network.

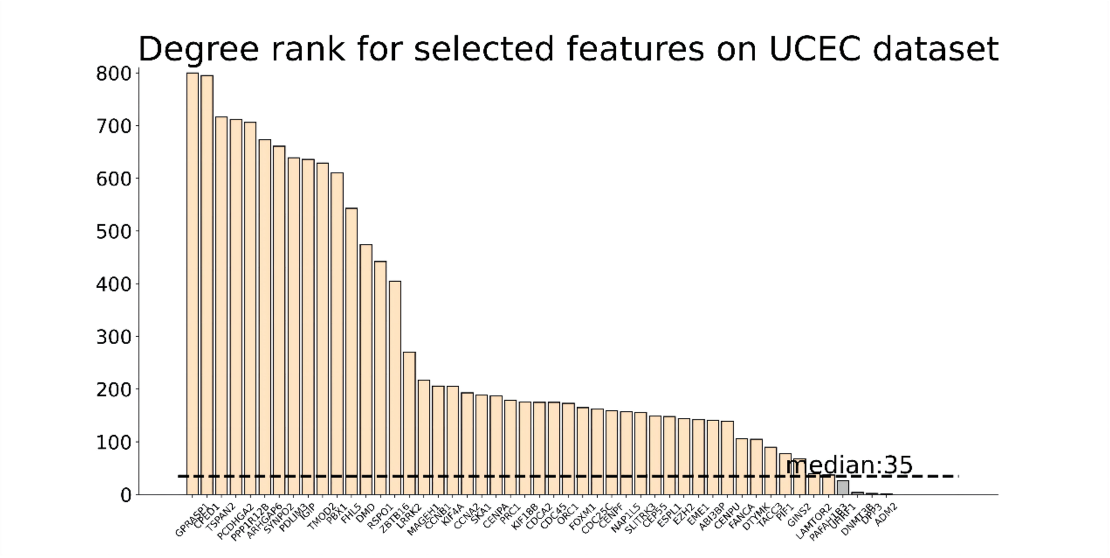

Figure S8. The degree of the features selected by EGFAFS for UCEC dataset in the differential co-expression network.

Figure S9-S16 show the results of GO enrichment on eight datasets (HNSC, LIHC, LUAD, LUSC, PRAD, STAD, THCA, and UCEC) with three aspects (BP, CC, and MF).

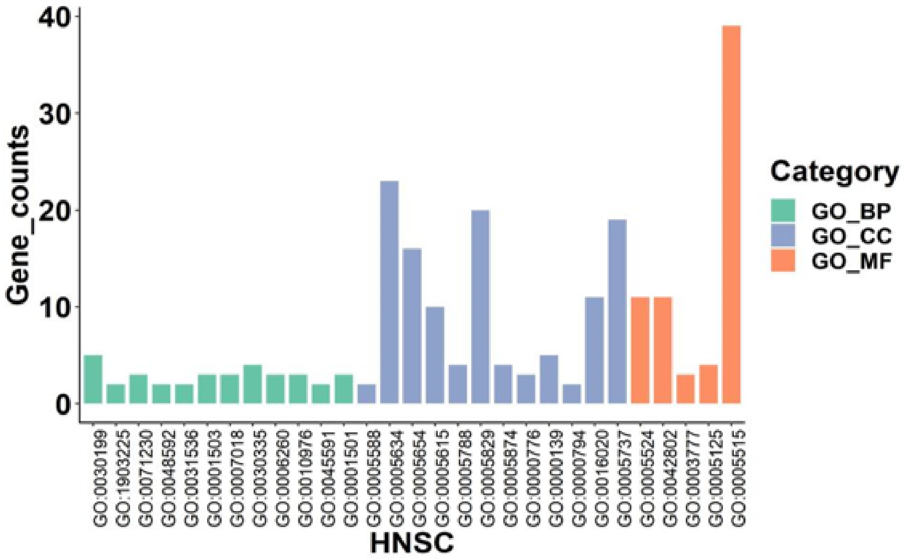

Figure S9. GO enrichment analysis of genes selected by EGFAFS on LIHC dataset.

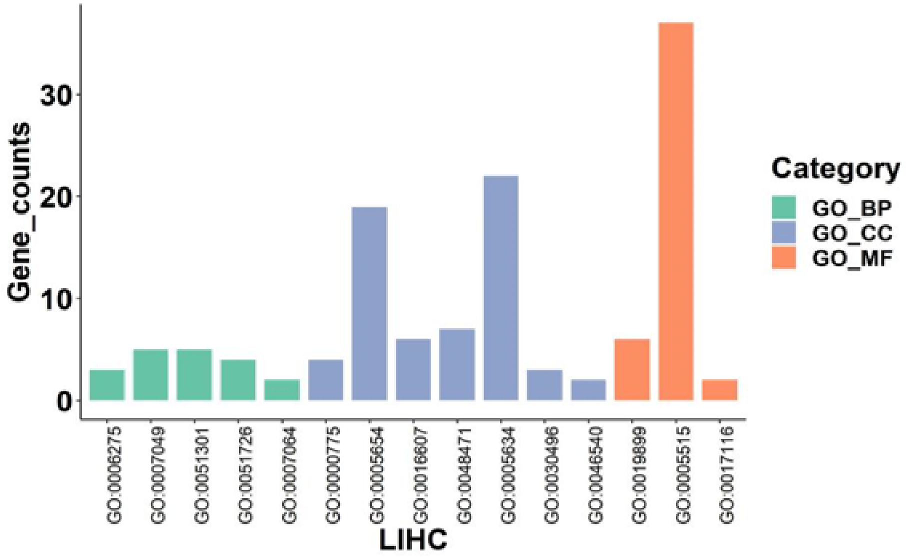

Figure S10. GO enrichment analysis of genes selected by EGFAFS on LIHC dataset.

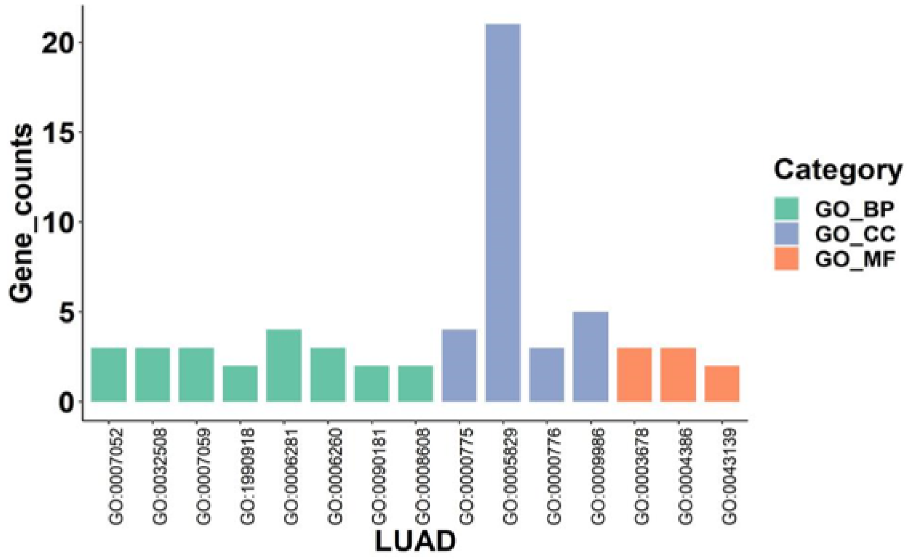

Figure S11. GO enrichment analysis of genes selected by EGFAFS on LUAD dataset.

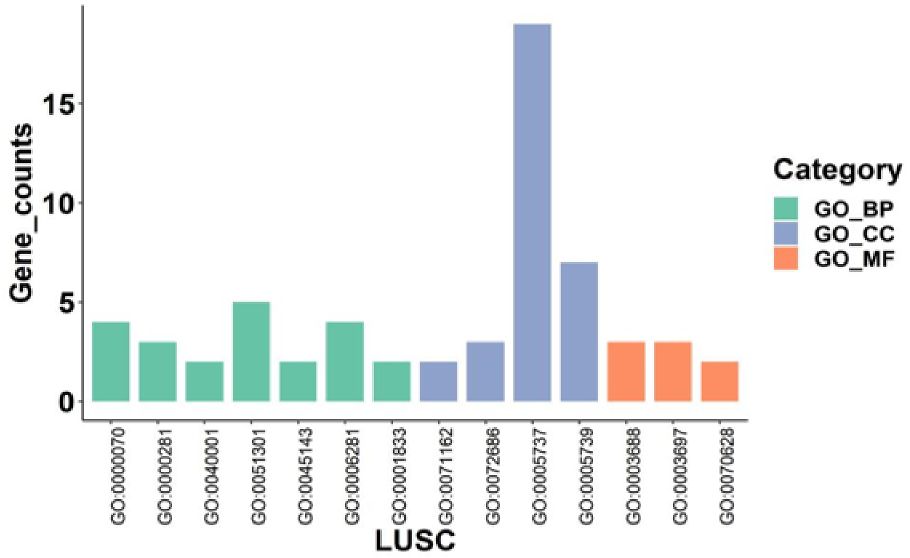

Figure S12. GO enrichment analysis of genes selected by EGFAFS on LUSC dataset.

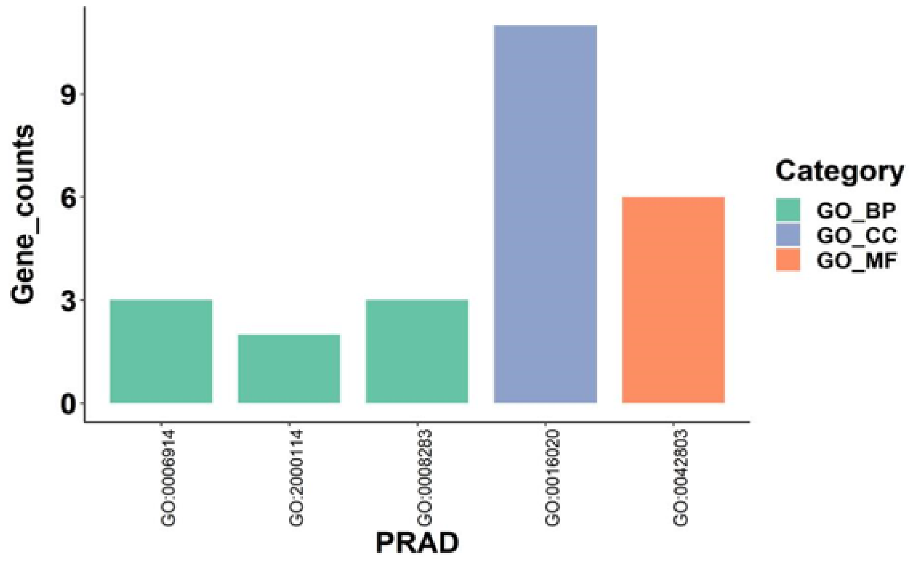

Figure S13. GO enrichment analysis of genes selected by EGFAFS on PRAD dataset.

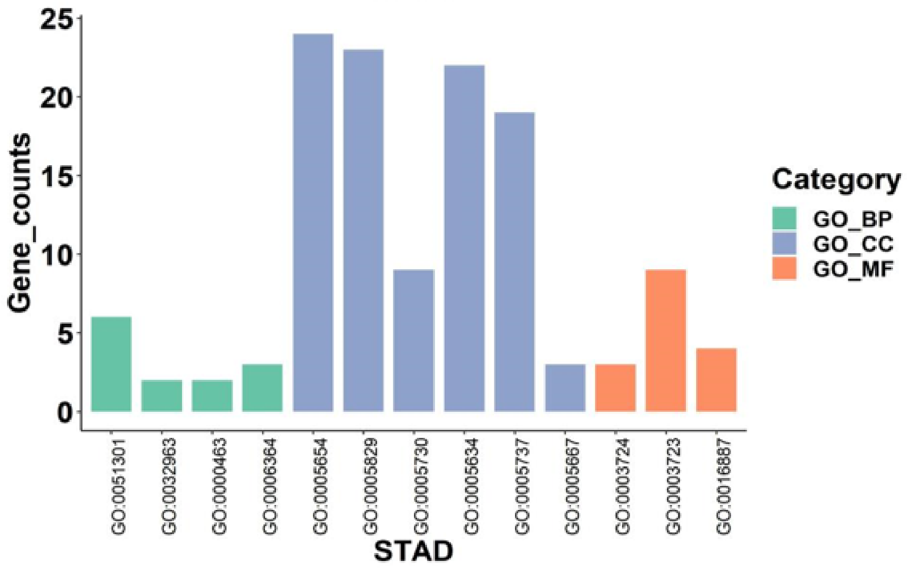

Figure S14. GO enrichment analysis of genes selected by EGFAFS on STAD dataset.

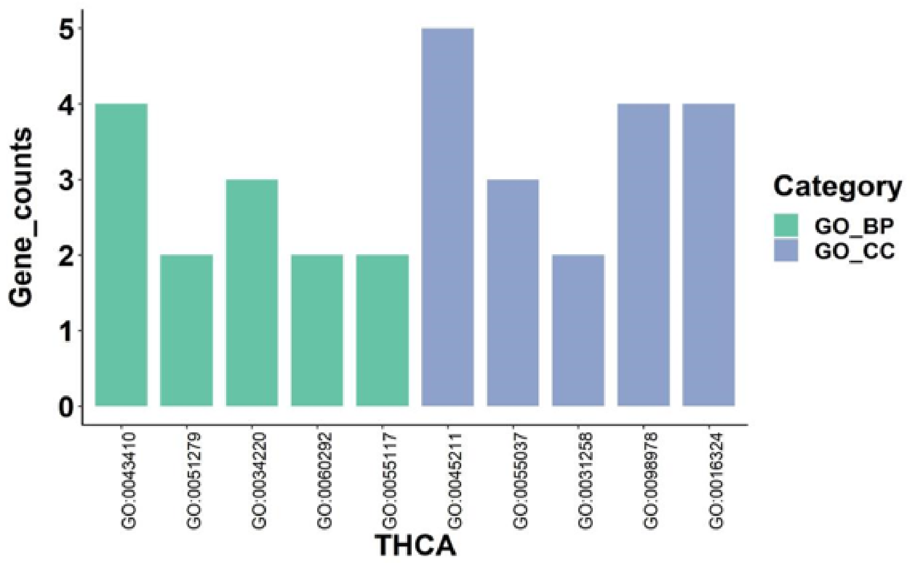

Figure S15. GO enrichment analysis of genes selected by EGFAFS on THCA dataset.

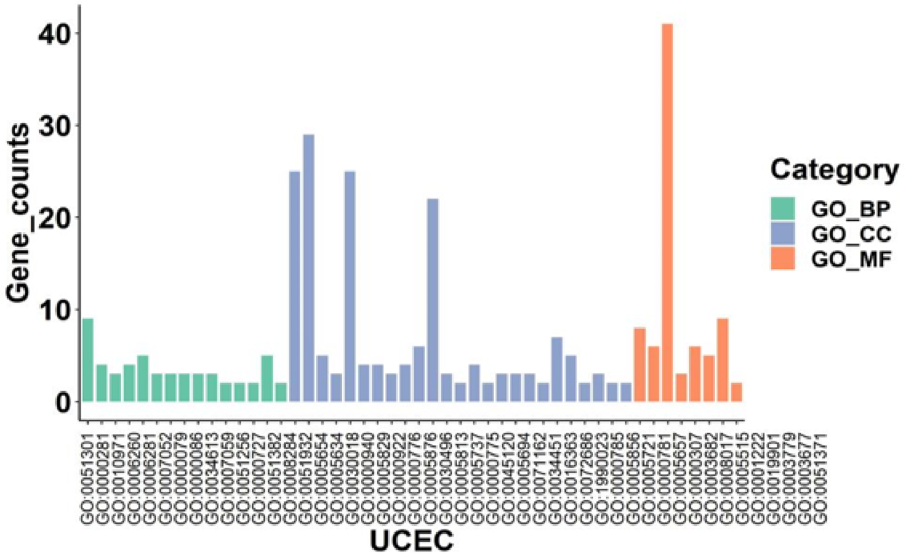

Figure S16. GO enrichment analysis of genes selected by EGFAFS on UCEC dataset.
